# Contribution of Patch Tests with Occupational Handled Products in the Diagnosis of Occupational Contact Dermatitis: A 10-year Review

**DOI:** 10.1155/2022/6768932

**Published:** 2022-08-08

**Authors:** A. Aloui, M. Maoua, S. El Guedri, A. Moussa, M. Bouhoula, A. Chouchene, I. Kacem, A. Brahem, N. Ghariani, H. Kalboussi, O. El Maalel, S. Chatti, M. Denguezli, N. Mrizek

**Affiliations:** ^1^Occupational Medicine Department, University Hospital of Farhat Hached, Faculty of Medicine of Sousse, University of Sousse, Sousse, Tunisia; ^2^Dermatology Departement, University Hospital of Farhat Hached, Faculty of Medicine of Sousse, University of Sousse, Sousse, Tunisia

## Abstract

**Introduction:**

Allergic contact dermatitis (ACD) is a common occupational disease. Its diagnosis is essentially based on interrogation and patch tests. However, commercially available batteries are sometimes not appropriate for the working conditions and the handled products, which must then be tested. In Tunisia, no previous study has focused on the contribution of patch tests with handled products in the workplace. The objective of this study is to establish the sociodemographic and occupational profile of the patients benefiting from patch tests with handled products in the workplace to identify the characteristics of these products as well as to evaluate the relevance of their positivity and their contributions in terms of aetiological diagnosis of occupational ACD.

**Methods:**

This is a retrospective descriptive epidemiological study conducted for a period of 10 years from January 1^st^, 2006, to December 31, 2015, among patients exercising a professional activity and consulting the Dermato-Allergology Unit of the Occupational Medicine ward of the University Hospital Farhat Hached of Sousse for the exploration of ACD.

**Results:**

During the study period, 113 patients received patch tests of handled products in the workplace with a prevalence of 7.3% of patch-tested patients during the same period. The mean age was 35.79 ± 9.45 years with a male predominance (sex ratio = 1.35). The most represented activity sectors were the health sector in 30.1% and the textile sector in 21.2%. The majority of patients were professionally active (61.9% of the study population) with an average professional seniority of 10.28 ± 8.49 months. In total, 138 patch tests with handled products were carried out of which 46 tests were positive (33.3%). After the analytical study, variables independently significantly associated with the positivity of patch tests with handled products in the workplace were the male gender and the working in the plastics industry. An occupational disease was declared to the National Health Insurance Fund for 8 patients, i.e., 7.1% of cases.

**Conclusion:**

Patch tests with handled products in the workplace can provide strong arguments for the professional origin of the ACD.

## 1. Introduction

An occupational dermatitis (OD) is a skin disorder exclusively caused or aggravated by work-related exposures [1]. In many countries, OD is the second most common occupational disease after musculoskeletal disorders [1, 2]. In Europe, occupational dermatitis account for 20 to 34% of occupational diseases [3]. In Tunisia, according to the statistics of the National Health Insurance Fund, OD accounted for 6.25% and 3.85% of all compensable occupational diseases in 2010 and 2012, respectively [4].

The most reported pathological form in industrialized countries is the contact dermatitis (CD) [5]. While irritant contact dermatitis represents the direct toxic effect of an offending agent on the skin found in 80% of CD, allergic contact dermatitis (ACD) represents a delayed-type hypersensitivity reaction (type IV) that occurs when allergens activate antigen-specific T cells in a sensitized individual observed in 20% of cases of CD [3, 6].

The etiological diagnosis of ACD is based on interrogation and patch tests. The commercially available series are not always adapted to the work conditions and to the products handled in the workplace. These products should therefore be tested, provided that their composition is known and they are correctly diluted, in order to avoid harmful effects, particularly caustic responses [7].

In this context, we carried out an epidemiological study on all the patients consulting the Dermato-Allergology Unit of the Occupational Medicine Department of the University Hospital Farhat Hached of Sousse for the exploration of ACD during the period from 2006 until 2015 to determine the sociodemographic and professional profile of patients patch-tested with handled products in the workplace and to identify the characteristics of these products as well as evaluating the relevance of the positivity of these tests and their contribution to the etiological diagnosis of occupational ACD.

## 2. Methods

This is a retrospective descriptive study conducted over a period of 10 years, from January 1^st^, 2006, to December 31, 2015, among all professionally active patients who consulted the Dermato-Allergology Unit of the Occupational Medicine Department of Farhat Hached University Hospital in Sousse (Tunisia).

We included all the data of patients who were patch-tested by the European Baseline Series (EBS) allergens (26 haptens until 2008 and then 28 haptens from 2008 to 2015) and the products handled in the workplace. Data were collected using a preestablished questionnaire covering sociodemographic and occupational characteristics and past illness history.

The patch tests were applied on the upper back of patients, using Finn Chamber patches. Test results were coded based on the intensity following the criteria from the International Contact Dermatitis Research Group [8].

In our study, we tested the handled products according to their nature: [7, 9–11].

- Irritant products: we resorted to a very low concentration of nonirritant dilution (1%; 0.01%; 0.001%).
- Textile products: a fragment of fabric (2*∗*2 cm dampened with saline solution) was applied to the patient's back skin during 48 hours.
- Plant products: plants were tested by their foliage, their stem, and their roots. Wood dusts were tested dispersed or not in Vaseline (10%).
- Gloves: both sides (external and internal) were tested.
- Cosmetic and hair products were tested dispersed in water (2%).
- Rubber products were diluted to a concentration of 1% in Vaseline and the achievement of a positive reaction was followed by the realization of a series of dilution. For resins, they were tested after extraction with acetone.
- Glues were tested with concentrations ranged from 10% to 100% in Vaseline. For the plaster, the test was performed on its external and internal sides.
- Greases were tested diluted or not in Vaseline.
- Water-soluble cutting fluids were tested diluted in water (1 to 50%) or pure as used by the worker.

Statistical analysis was done using SPSS software. The *p* value threshold was set to 0.05.

## 3. Results

Among all the patients consulting the Dermato-Allergology Unit of the Occupational Medicine Department of Farhat Hached University Hospital in Sousse (1544 patients) during the study period, 113 patients had benefited from patch tests with products handled in the workplace presenting a prevalence of 7.3%.

The mean age was 35.79 ± 9.45 years. A male predominance was noted (65 men versus 48 women) with a sex ratio of 1.3 (75.2%). The majority of patients were employed in the healthcare sector (30.1% of cases) and the clothing sector (21.2% of cases) (Table 1). The average job tenure was 10.28 ± 8.49 months. Only 54 patients, i.e., 47.8% of the cases, had extraprofessional activities, predominated by housekeeping (39 cases i.e. 34.5%). A personal history of allergy (both cutaneous and noncutaneous) was noted in 27.4% of cases. Allergic rhinitis was observed in 12.4% of cases.

The hands were the most affected site in 74.3% of cases, followed by the forearms (16.8% of cases) and the face (15.9% of cases). The clinical aspect was polymorphic in 97 patients (85.8%). Indeed, more than half of our patients (63 cases, i.e., 55.8%) had erythematous-vesicular lesions, whereas 34 patients (30% of cases) had erythematous-squamous lesions.

Almost all of the patients (*n* = 112, i.e. 99.1%) were patch-tested using the European baseline series (EBS). The most frequent allergens were metals (Nickel in 17%, Cobalt in 16.1% and chromium in 15.2%). The EBS allergen patch test results are summarized in Table 2.

A total of 138 patch tests to the handled products of different nature were performed (Table 3). These tests were positive in 46 cases and this positivity was found in 36 patients (33.33% of the cases) because some patients were positively tested to 2 or 3 handled products simultaneously.  Table 4 shows the sensitization of our patients to handled products in the workplace. All positive patch tests to handled products were relevant to the current allergic episode. Patch tests to EBS allergens were negative in 19 patients (16.8%) while the patch test to the handled products was positive. A declaration of an occupational ACD was proposed for 8 patients (7.1%).

The univariate analysis allowed concluding that several factors were significantly associated to the positivity of the handled products in workplace such as the male gender, the sectors of plastic and painting, the personal history of allergic rhinitis, certain localizations (cheeks and forearms), the erythematous aspect, and the positive reaction to rosin (Table 5).

After multivariable logistic regression, the statically significant independent variables associated with the positivity of patch tests to handled products were male gender and plastics sector (*p*=0.023, Ora = 2.83 (1.1–6.9) and *p*=0.04, Ora = 10.29 (1.1–95), respectively).

## 4. Discussion

ACDs are one of the most common occupational diseases. Thus, we collected all the data of patients who were patch-tested by the EBS allergens and the handled products in the workplace, realized in the Dermato-Allergology unit of the Occupational Medicine department of the Farhat Hached University Hospital in Sousse in order to describe the socio-demographic and professional profile of these patients, to identify the characteristics of these products and to evaluate the relevance of the positivity of these tests and their contribution in terms of the etiological diagnosis of occupational ACD. During the study period, 113 patients had benefited from patch-tests with products handled in the workplace presenting a prevalence of 7.3%.

The mean age of our population was 35.79 ± 9.45 years. However, in the literature, age did not seem to influence the positivity of patch tests to the handled products. Some authors suggest that occupational ACD can occur at any age but it mostly affects young subjects with a mean age of 22 years in women and 31 years in men [12].

A male predominance was noted in our study (57% men versus 43% women) which aligned with the results found by Schwensen et al. [13] in their survey among 1000 cases of occupational ACD (61.8% men versus 38% women). Our results suggested that male gender was significantly associated with the positivity of patch tests to handled products in the workplace (*p*=0.01). However, in the study of Slodownik et al. [14], the population was predominantly male (71%) but gender did not seem to influence the results of patch tests with the handled products (*p*=0.89).

In our study, hands, forearms, and face were the most common locations with 67.2%; 18.6%, and 17.7% of cases, respectively, which is similar to the majority of studies' results. Indeed, Raison-Peyron [15] described that occupational dermatitis predominated on the dorsal side of the fingers, hands, and wrists. A study conducted in Australia had shown that the hands were the primary site for occupational ACD lesions (70.2% of cases) followed successively by the forearms (20.2%) and the face (19.6%) [16].

The healthcare sector (30.1% of cases) and the clothing sector (21.2% of cases) were the most provider sectors of ADC in our population. This can be explained by an ascertainment bias due to the proximity of the University Hospital of Farhat Hached to an industrial zone specialized in textiles and the consultation of healthcare personnel. The plastics sector was significantly associated with the positivity of patch tests with handled products in the workplace and this association persisted even multiple binary logistic regression (*p*=0.012).

Patch tests are an essential diagnostic tool in dermato-allergology and consist of occlusively applying various allergens to an intact part of the skin [17]. The most conventional allergens are collected in test series, such as EBS and other additional series [18]. The EBS patch test recommended by the International Contact Dermatitis Research Group (ICDRG) [19], were performed on 112 patients within our study population (99.1%) of which 46.4% of cases had a positive response. The positivity of a patch test with EBS allergens may reflect only immunological sensitization, without the allergen being responsible for the symptoms. Thus, patch tests with the handled products in the workplace are justified in this context in order to distinguish several allergens involved, which cannot be substituted in the workplace, or to highlight the responsibility of a new allergen not yet described.

In our study, the only EBS allergen significantly associated with a positive patch test to handled products in the workplace was rosin/colophany. These professional products were glues and plastic products in an automotive industry worker. In the literature, the most common reactions were epoxy resins (24.7%) followed by thiuram (16.9%) and rosin (13.0%). Of those who reacted to rosin, 50% were traders. The majority of those who reacted to epoxy resins were also traders (84.2%) [14].

In our survey, 138 patch tests to the products handled were performed, 46 were positive (33.33% of the cases) among 36 patients. Nineteen patients had a positive patch test while EBS was negative. In a study done in Australia [14], among 1532 participants, 101 (6.6%) patients reacted to their own products. In a German study of the IVDK network [20], among 2460 patients who were patch-tested with their manipulated products between 1989 and 1992, 208 (8.5%) had a positive reaction. Relevant tests were noted in 44% of these cases. The substances tested and showing positive reactions were medical products (45%), cosmetics (39.4%), rubbers (4.1%), and leather products (0.7%).

The products handled in the workplace are very varied and their number is constantly growing.

Plastics are ubiquitous. They are a common cause of occupational ACD especially in the plastics industry [21]. In our population, 7 patch tests with handled plastic products (thermoset plastics, resins, gloves, etc.) were carried out, of which 57.2% were positive. Goossens et al. [22] tested 15141 patients from 1978 to 2001 and diagnosed occupational ACD related to exposure to plastic products in 26 patients [21].

ACD in textile products is usually caused by clothing or fabrics handled in a professional environment [23]. In our survey, 33 patch tests with textile handled products were carried out of which 18.1% were positive. Among them, two patients had a positive patch test for work clothes, one had negative EBS patch test. Of the three patients with a positive patch test for tissue handled in the workplace, one patient had a negative EBS patch test, one was allergic to nickel and sesquiterpene lactone, and one patient to whom EBS testing was not performed due to lack of products to be tested. The patient with a positive patch test for leather gloves had negative EBS patch test.

Cosmetics are a common cause of contact dermatitis due to the presence of fragrances and preservatives [24]. In our study, among the 6 patch tests to cosmetic products (hair products and primer gel) that were performed, 4 were positive. The patient with a positive primer gel test had negative EBS patch test. The three patients with positive hair product tests had EBS patch tests positive to nickel and chromium. Sostedet al. [25] published in 2004 a study on the sensitizing power of different hair dyes and identified 229 potentially sensitizing substances of which 75% are considered moderate to strong allergens and only 5 of these substances are available in patch tests.

According to the scientific and epidemiologic researches, some authors have cited some indications for patch tests with handled products in the workplace:

  Diagnosis confirmation of ACD, when discrepancies occur between a patient's clinical signs and patch-test results [26]
  Etiological diagnosis of ACD when the patient is sensitized to multiple allergens and it is necessary to determine those that have clinical significance [26]
  The etiological diagnosis of occupational dermatological allergies, in order to establish a link between pathology and occupational exposure, with the aim of a possible job adjustment or recognition as an occupational disease [27]
  The exact composition of the products manipulated by the employee the workplace is not fully known [27]

Our original study is one of the rare survey dealing with patch-tests with handled products However, it admits some limitations. The retrospective nature of our study, which is based on a pre-established medical history sheet, and some socio-professional and medical data may be missing from these records. Data on workplace products provided by the employee and safety data sheets may be insufficient. Some handled products in the workplace may be unknown, which leads to an underestimation of these occupational dermatoses. However, the interrogation, clinical examination, the patch-tests lecture were carried out by experienced specialists using the same material which reduces the sources of errors related to methodological heterogeneity.

## 5. Conclusion

This study has shown that patch tests with handled products in the workplace provide solid arguments in favor of the professional origin of ACD. For 19 patients, tests for manipulated products had added value since the EBS was negative. With the appearance of new professional agents leading to the increased prevalence of ACD, the impact on productivity is continuously growing. Thus, the need to introduces adequate preventive measures through two components: technical and medical prevention.

## Figures and Tables

**Table 1:** Distribution of the study population according to work tasks.

Work task	Number (*n*)	Percentage (%)
	34	30
Textile industry	24	21, 3
Automotive industry	8	7
Metallurgical industry	8	7
Plastic industry	6	5, 3
Welding industry	4	3, 5
Administration industry	3	2, 7
Police	3	2, 7
Cleaning industry	3	2, 7
Paint industry	2	1, 8
Wood industry	2	1, 8
Hairdressing industry	1	0, 9
Electric industry	1	0, 9
Other industries	14	12, 4
Total	113	100

**Table 2:** Results of EBS allergen patch tests.

Allergens	*Sensitization*			
	*Yes*		*No*	
	Number	Percentage (%)	Number	Percentage (%)
Nickel	19	17	93	83
Cobalt chloride	18	16, 1	93	83
Potassium dichromate	17	15, 2	94	84, 8
Thiurams mix	11	9, 8	101	90, 2
IPPD	6	5, 3	106	93, 7
Fragrance mixII	6	5, 4	106	94, 6
Fragrance mixI	5	4, 5	107	95, 5
Benzocaine	3	2, 7	109	97, 3
Peru balsam	3	2, 7	109	97, 3
Lanolin	3	2, 7	109	97, 3
Paraben mix	3	2, 7	109	97, 3
Kathon CG	3	2, 7	109	97, 3
Lactones	3	2, 7	109	97, 3
Epoxy resin	3	2, 7	109	97, 3
Dibromo-dicyanobutane	3	2, 7	109	97, 3
Mercapto mix	2	1, 8	110	98, 2
Quaternium 15	2	1, 8	110	98, 2
Rosin	2	1, 8	110	98, 2
HMPCHC	2	1, 8	110	98, 2
Formaldehyde resin	1	0, 9	111	99, 1
Primine	1	0, 9	111	99, 1
PPD	1	0, 9	111	99, 1
Formaldehyde	1	0, 9	111	99, 1
Neomycin	1	0, 9	111	99, 1
Mercapto benzothiazole	0	0	112	100
Clioquinol	0	0	112	100
Budesonide	0	0	112	100
Tixocortol pivalate	0	0	112	100

**Table 3:** Distribution of handled products.

Handled product	Examples
Plastic products	(i) Thermosetting polymer
	(ii) Resins
	(iii) Gammex gloves
	(iv) Nitrile gloves

Plant products	(i) Latex gloves
	(ii) Wood dust
	(iii) Plants
	(iv) Flour

Textile	(i) Fabric handled
	(ii) Work clothes
	(iii) Red and black leather
	(iv) Leather gloves

Rubber additives	(i) Rubber gloves
	(ii) Rubber cables
	(iii) Rubber gum

Metals	(i) Welding products

Oils	(i) Natural oils
	(ii) Paraffin oil
	(iii) Cutting oils
	(iv) Engine oils
	(v) Industrial grease

Detergents	(i) Detergents

Solvents	(i) Solvents

Biocidal	(i) Disinfectant

Cosmetics	(i) Primer gel
	(ii) Hair products

Glues	(i) Glues
	(ii) Plaster

Fuel	(i) Petrol and diesel

Other	(i) Diluted liquid soap
	(ii) Softener
	(iii) Polyamine salts
	(iv) Jeans washing products
	(v) Verner flash

**Table 4:** Results of patch tests with products handled in the workplace.

Products handled	*Sensitization*		
	Number (*n*)	Yes	No
Latex gloves	34	8	26
Fabric handled	19	3	16
Work attire	12	2	10
Cutting oil	10	2	8
Rubber gloves	7	5	2
Hair products	5	3	2
Glue	5	2	3
Grease	5	1	4
Diluted liquid soap	3	0	3
Detergent	3	1	2
Cleaning product	3	1	2
Nitrile gloves	2	1	1
Resins	2	1	1
Welding products	2	1	1
Thermosetting polymers	2	2	0
Wood dust	2	1	1
Vernier flash without dilution	2	1	1
Vernier flash with dilution	1	0	1
Rubber cables	1	1	0
Cotton	1	0	1
Flour	1	1	0
Disinfectant	1	1	0
Plants	1	0	1
Gloves gammex	1	0	1
Leather gloves	1	1	0
Solvent	1	1	0
Red and black leather	1	0	1
Petrol and diesel	1	1	0
Jeans washing products	1	1	0
Primer gel	1	1	0
Rubber gum	1	0	1
Polyamine salts	1	1	0
Engine oil	1	0	1
Band-aid	1	1	0
Paraffin oil	1	0	1
Natural oils	1	1	0
Softener	1	0	1
Total	138	46	92

**Table 5:** Results of patch-tests with handled products and variables of interest.

	*Test result of patch test with product handled in the workplace*		*p*
	Negative	Positive	
*Gender*			
Men (*n* (%))	38 (49, 4)	27 (75)	0, 01
Women (*n* (%))	39 (50, 6)	9 (25)	

Mean age (years ± SD)	35.52 ± 9.58	36.36 ± 9.27	0, 661

Professional seniority (years ± SD)	10.52 ± 8.76	9.78 ± 8.01	0, 67

*Allergic rhinitis*			
Yes	13	3	0, 034
No	64	33	

Location: Cheeks	5	7	0, 037

Location: Forearm	9	10	0, 03

*Erythematosus appearance*			
Yes	19	3	0, 041
No	58	33	

*Erythematosicular appearance*			
Yes	40	23	0, 23
No	37	13	

*Sensitization to dichromate*			
Positive	12	5	0, 85
Negative	65	30	

*Sensitization to nickel*			
Positive	13	6	0, 97
Negative	64	29	

*Sensitization to cobalt*			
Positive	14	31	0, 36
Negative	63	4	

*Sensitization to rosin*			
Positive	2	1, 8	0, 034
Negative	110	98, 2	

Plastics sector	108	5	0, 012

Painting sector	111	2	0, 037

## Data Availability

The data were collected from a medical document of the occupational department.
